# Predicting medical students who will have difficulty during their clinical training

**DOI:** 10.1186/s12909-017-0879-2

**Published:** 2017-02-21

**Authors:** D. L. Jardine, J. M. McKenzie, T. J. Wilkinson

**Affiliations:** Department of General Medicine, Christchurch Hospital, University of Otago, Riccarton Ave 2, Christchurch, 8011 New Zealand

## Abstract

**Background:**

We aimed to classify the difficulties students had passing their clinical attachments, and explore factors which might predict these problems.

**Methods:**

We analysed data from regular student progress meetings 2008–2012. Problem categories were: medical knowledge, professional behaviour and clinical skills. For each category we then undertook a predictive risk analysis.

**Results:**

Out of 561 students, 203 were found to have one or more problem category and so were defined as having difficulties. Prevalences of the categories were: clinical skills (67%), knowledge (59%) and professional behaviour (29%). A higher risk for all categories was associated with: male gender, international entry and failure in the first half of the course, but not with any of the minority ethnic groups. Professional and clinical skills problems were associated with lower marks in the Undergraduate Medical Admissions Test paper 2. Clinical skills problems were less likely in graduate students.

**Conclusions:**

In our students, difficulty with clinical skills was just as prevalent as medical knowledge deficit. International entry students were at highest risk for clinical skills problems probably because they were not selected by our usual criteria and had shorter time to become acculturated.

## Background

Much has been written on the validity of various selection criteria for medical students [1–4]. These studies have looked at correlation between results from selection criteria (eg academic grades and cognitive tests) and examinations later in the training program [5, 6]. Correlation between these criteria and medical school assessments weakens as students progress through clinical training [4, 7]. For some interview assessments, correlations may strengthen during clinical training but results are limited at the outset by interviewer reliability [8, 9]. Most studies of this type look at predicting success in medical school.

Finding reliable criteria specifically for predicting students who might struggle is is not as well researched, and cannot be assumed to simply be the converse of those which predict success [10]. Also, finding a lack of correlation between selection criteria and medical school assessments does not inform us as to what part of the clinical performance is not predicted. Furthermore, many prediction studies have used results at the end of clinical training as end-points but from a teaching perspective it is very important to seek out “at risk” students during the first months of clinical training. Students who perform well before selection will usually continue to perform well during clinical training, irrespective of their teaching, whereas those who struggle consume disproportionate amounts of teacher time and may not even complete the course [1]. There are gaps in the literature around predicting who might struggle. Filling such gaps is likely to inform student selection policies. Our study aims to look at what (if any) predictors are potentially useful.

The assessment programme at the University of Otago, Christchurch, is designed to systematically identify at risk students [11]. This system set out deliberately to be sensitive, rather than specific. In other words, it aimed to detect all students who might be at risk, in the knowledge that some were not really at risk rather than miss students who could benefit from assistance. Over the time prior to our study the “ethnicity mix” of our medical students changed with some using English as their second language [12–14] Associated with this trend, the University of Otago Medical School temporarily increased the numbers of full-fee paying international students directly into years 2 and 4 of our 6-year course. Importantly, these students were mainly government-sponsored and did not undertake the usual entry pathway that includes a Health Sciences First Year 728SFY] course. Therefore they by-passed the standard selection criteria including the first year Grade Point Average (GPA) and Undergraduate Medical Admissions Test or UMAT [6]. We formed the impression that they were having problems progressing through years 4 to 6 and that this was mainly because of difficulties with communication during history taking.

We aimed to clarify how many students were having difficulty within their clinical attachments, what the major areas of difficulty were, and whether these students could be predicted by various demographic variables, selection criteria and previous academic assessments. We hypothesised that significant numbers of students were at risk because of clinical skills issues (including communication) and that this might relate to their selection pathway.

## Methods

### Course and context

**Fig. 1:**
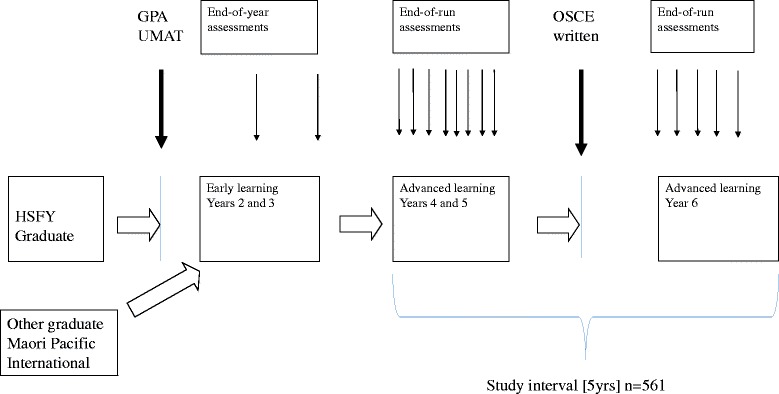
Selection, medical training course and study interval. Timeline showing pathways leading to selection into medical school and progress through early and advanced learning courses [unfilled arrows]. Vertical arrows refer to end-of-run and end-of-year assessments; bold arrows refer to major examinations for selection and end-of-5^th^ year “finals”. GPA = grade point average, UMAT = Undergraduate Medical Admissions Test, OSCE = Objective Structured Clinical Examination, written = multi-choice and short answer examinations, HSFY = Health Sciences First Year, Other graduate refers to students with allied-health degrees. International refers to full-fee-paying students from overseas. Other graduates, Maori and Pacific students, and International students are exempt from the standard GPA- UMAT selection process

The main selection pathway into year 2 classes at Otago Medical School uses a combination of Grade Point Average (GPA) from performance during the HSFY at Otago University, and the UMAT (Fig. 1). The UMAT (used in Australia and New Zealand) is a multi-choice test divided into 3 papers: logical reasoning and problem solving; understanding people, and non-verbal reasoning [6]. Competitive graduate entry (based mainly on GPA for their degree and less on UMAT) is available for approximately 20% of students. In addition there are “other” pathways for the selection of special groups, for example allied health graduates, international graduates, Maori and Pacific students all of whom are interviewed and require a reasonable GPA standard. Some of these students sit UMAT but the results are not used for selection purposes. Finally there are full-fee paying international students who are selected into year 2 or year 4 classes on the basis of University marks in their country of origin. For all applicants, the selection process does not include any observed assessment of the student interacting with others. Thereafter, the course is divided into “early learning” years 2 and 3 (mostly professional foundations and biomedical sciences), and “advanced learning” years 4, 5 and 6 (mostly clinical). Years 4–6 are undertaken at one of three campuses. All students at one campus, formed the context for this study where approximately 80 students embark on three years of clinical training each year. The clinical teachers at this campus are not involved in the “early learning” course and do not have information relating to previous academic performance. During years 4 and 5 they rotate through a variety of 8-week clinically-based “block modules” simultaneously with vertical modules which run throughout the course. At the end of year 5, all students are required to pass three written papers and one final Objective Structured Clinical Examination [OSCE] before progressing to year 6. To be eligible to sit the examinations at the end of year 5, students must, with very few exceptions, pass all of their modules. For each block and vertical module, the module convenors have documented and publicised “essential criteria” for achieving a pass. Essential criteria usually include what are considered basic clinical skills such as history taking, physical examination, interpretation of results, and well-defined professional behaviours. Formal assessments occur during or at the end of all block modules. Judgements on clinical skills are made using a combination of assessment methods – typically mini-CEX or OSCE-style direct observations under examination conditions. In many attachments, students are also required to write a clinical record of patients they have seen and these are marked using documented criteria. Knowledge is tested using combinations of MCQ or short-answer papers, oral examination and essays on a subject of interest chosen by the student. Professional behaviour is assessed on the basis of a number of publicised criteria. [11, 15]. A student is awarded a “conditional pass” (CP) if they neither clearly fail nor pass a clinical assessment. For all conditional passes the conditions required to achieve a pass (eg remedial teaching sessions, repeat and pass a clinical assessment) are clearly stated [11]. This is consistent with our desire for a programmatic approach to assessment and for the detection system to be sensitive rather than specific. A student who subsequently does not achieve the condition(s) is awarded a “fail” (F) for the attachment. A student who achieves the condition(s) is given a “pass after conditions met” (PACM). The awarding of a CP or F automatically results in the student being discussed at quarterly “student progress” meetings which are attended by all attachment convenors and chaired by the Dean.

### Data collection

Basic demography and previous academic results were accessed from data already collected by the University following admission to medical school. These data included ethnicity, defined as the cultural group the student felt affiliated to. Ethnic groups were Pakeha (New Zealand European), Māori (New Zealand indigenous), Pacific, Asian, Indian and “others” (which included African, Middle-East and European). For all of these groups we did not have specific data on (a) their “first” language and (b) duration of time spent in NZ prior to medical school entry. We also collected data on selection pathway, with particular reference to whether or not UMAT was part of the process, UMAT performance (on all 3 papers), GPA and academic progress through years 2 and 3.

To identify the students who were having difficulties during clinical training, we retrospectively analysed data collected for all year 4–6 students discussed at student progress meetings over a 5-year period between 2008–2012 inclusive. Over this study interval, all students who progressed through some or all of their clinical training years at the study campus were designated the “total group” (*n* = 561). The students who progressed through uneventfully (without CP’s or F’s) were designated the “control group” (*n* = 358). Students who were awarded a CP or F for any assessment comprised the “study group” (*n* = 203). Assessments were clustered into three broad problem categories: knowledge, professional behaviour and clinical skills, and the proportions of students in each category were calculated (Table 1). The clinical skills category was made up predominantly by communication skills in history taking . The clustering was undertaken by each of the authors independently and discrepancies discussed until consensus was reached. Note that the total number of CP’s across all attachments (*n* = 374) far exceeded the number of students in the study group (*n* = 203) because several were awarded CP’s in more than one assessment. As we wished only to identify any student with difficulty, students were not classified according to how many CP’s they were awarded. Similarly, the numbers of students awarded a CP in each assessment did not add up to the numbers in each category because students awarded CP’s in multiple assessments were only counted once. Furthermore, for the risk analysis, the likelihood of being awarded a CP was dependent on the number of years the student spent in the study period: some students were observed for all three of their clinical years while others were observed for fewer. To account for these varying periods of risk we used Cox regression analysis, using the category of problem as the outcome, the years observed as the time variable, and the various demographic and assessment variables as predictors. For each we calculated the hazard ratios (HR) and 95% confidence intervals. This analysis did not allow for interactions between some of the demographic variables, (for example age and graduate entry).

**Table 1:**
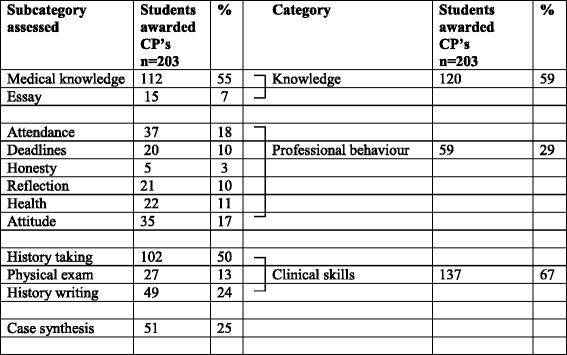
Numbers of students awarded conditional passes in subcategories and categories of assessment in clinical training

Students awarded CP’s refers to the number of students awarded conditional passes in any sub-category assessment during their clinical training. For categories, students with CP’s in more than one subcategory were counted only once

We obtained ethics approval for tracking student outcomes based on selection measures, of which this study is a subcomponent. Furthermore this was an observational study and individual and identifiable students’ results cannot be discerned from the data.

## Results

Over the five-year study interval (2008–2012) the average time interval over which data were gathered from students was 2.4 years (median 2.0; range 1–4). Out of the total group (*n* = 561), 203 students were awarded one or more CPs or F’s during the study interval and comprised the “study group” of at risk students. In total we awarded 374 CP’s and 64 fails. As expected, far more of the study group were awarded CP’s in years 4 and 5 (*n* = 198, 98%) than in year 6 (*n* = 48, 24%). In year 6, only 7 students were awarded their first CP. As a consequence of these results, 10 students in the study group failed the in-course component and were not permitted to sit the end-of-year examinations. They therefore repeated the year or withdrew from the course. A further 8 students failed the end-of-year 5^th^ year examinations.

The most frequent assessments contributing to CP’s were (in decreasing order of frequency): medical knowledge (55% of students in the study group), observed history taking (50%), case synthesis (25%) and history writing (24%) (Table 1). When the assessments for history taking, history writing and physical exam were combined to make up the clinical skills category, this became the most prevalent problem in the study group (67%), ahead of knowledge (59%) and professional behaviour categories (29%).

Hazard ratios for variables that might predict each problem category and failure of a year (and end-of-year examinations) are shown in Table 2. Male students (41% of the total group) had a higher risk of being awarded a CP for all categories: knowledge HR = 1.45 (1.01-2.08 *p* = 0.04), professional behaviour HR = 3.45 (1.96-5.88, *p* < 0.001) and clinical skills HR = 2.04 (1.47-2.86, *p* < 0.001). International students starting in year 2 classes had over twice the risk of a CP for clinical skills HR = 2.71 (1.91-3.83, *p* < 0.001) and knowledge: HR = 2.43 [1.68-3.51, *p* < 0.001]. International students starting in year 4 were at similar risk: HR = 2.37 (1.44-3.91, *p* < 0.001) and 2.51 (1.5-4.18, *p* < 0.001) for clinical skills and knowledge respectively.

By contrast, minority ethnic groups were not at risk for any problem category and graduate entry students had half the risk of being awarded a CP for clinical skills: HR = 0.47 (0.25-0.87, *p* = 0.02). Students who scored higher marks in UMAT paper 2 (“understanding people”) had lower risk of a CP for professional behaviour: HR = 0.88 (0.98-1.0, *p* = 0.03) and clinical skills: HR = 0.90 (0.98-1.0, *p* = 0.01). Not surprisingly, students with a higher GPA average had lower risk of a CP for knowledge: HR = 0.95 (0.92-0.98, *p* = 0.001). Students who failed or repeated years 2 and 3 had over twice the risk of a CP for knowledge: HR = 2.52 (1.27-5.03, *p* = 0.01) and 2.16 (1.08-4.29, *p* = 0.03) and clinical skills: HR = 2.78 (1.49-5.18, *p* = 0.001) and 2.38 (1.27-4.44, *p* = 0.01); and over 3 times the risk of a CP for professional behaviour: HR = 4.17 (1.75-10.0) and 3.58 (1.50-8.55, *p* = 0.001).

Failing a clinical year was associated with younger age: HR = 1.13 (1.00-1.28, *p* = 0.02), entry without sitting UMAT: HR = 2.89 (1.51-5.48, *p* = 0.01), international entry into years 2 and 4: HR = 2.35 and 3.11 (1.32-4.19 and 1.26-7.70, *p* = 0.001 and 0.01), and failure in year 2: HR = 3.82 (1.13-9.00, *p* = 0.03).

**Table 2:** Student demography and preclinical performance as predictors for problems in clinical training

	n		Knowledge problem				Professionalism problem				Clinical skills problem				Fail year or EOY exam			
	N	Y	HR	95% CI	95% CI	*p*	HR	95% CI	95% CI	*p*	HR	95% CI	95% CI	*p*	HR	95% CI	95% CI	*p*
Age		560	1.05	0.96	1.15	0.25	1.11	1.00	1.24	0.05	0.98	0.89	1.08	0.70	1.13	1.00	1.28	0.02
Female Gender	229	332	0.69	0.48	0.99	0.04	0.29	0.17	0.51	<0.001	0.49	0.35	0.68	<0.001	0.50	0.27	0.93	0.15
European ethnicity	259	304	1.43	.99	2.07	0.06	1.13	0.67	1.90	0.65	1.18	0.84	1.67	0.33	1.03	0.56	1.90	0.93
Maori ethnicity	536	27	0.54	0.17	1.70	0.29	1.66	0.60	4.62	0.33	0.98	0.43	2.23	0.97	0.61	0.08	4.46	0.63
Asian ethnicity	359	204	0.88	0.60	1.28	0.50	1.03	0.60	1.75	0.93	0.84	0.59	1.21	0.36	0.90	0.47	1.70	0.78
Indian ethnicity	546	17	0.60	0.19	1.88	0.38	0.40	0.06	2.90	0.37	0.90	0.37	2.20	0.82	0.51	0.07	3.73	0.51
Other ethnicity	563	30	0.70	0.26	1.90	0.48	0.77	0.19	3.14	0.71	0.94	0.42	2.14	0.89	2.58	0.91	7.33	0.08
Entry pathway that did not require UMAT	396	165	2.00	1.40	2.88	<0.001	1.45	0.85	2.46	0.18	1.61	1.14	2.26	0.01	2.89	1.52	5.48	0.00
Graduate entry	461	100	0.85	0.51	1.43	0.55	0.84	0.40	1.75	0.64	0.46	0.25	0.85	0.01	0.65	0.23	1.85	0.42
Entry after first year at University	147	414	0.66	0.45	0.95	0.03	0.95	0.54	1.69	0.87	0.88	0.61	1.27	0.49	0.82	0.41	1.66	0.59
International student entering Y2	490	74	2.47	1.54	3.95	<0.001	0.98	0.50	1.92	0.95	3.19	1.97	5.18	<0.001	2.35	1.32	4.19	<0.01
International student entering Y4	539	25	4.93	2.11	11.49	<0.001	2.79	1.18	6.58	0.02	4.24	1.81	9.90	<0.01	3.11	1.26	7.70	0.01
UMAT paper 1 for each 10% change in mark		396	0.91	0.81	1.02	0.09	0.90	0.78	1.05	0.17	0.93	0.84	1.03	0.18	0.86	0.72	1.05	0.13
UMAT paper 2 for each 10% change in mark		396	0.94	0.86	1.04	0.21	0.88	0.77	0.99	0.03	0.90	0.83	0.98	0.01	0.85	0.72	1.02	0.15
UMAT paper 3 for each 10% change in mark		396	1.06	0.94	1.18	0.34	1.06	0.99	1.23	0.47	1.05	0.95	1.16	0.36	1.12	0.91	1.39	0.29
GPA		450	0.95	0.92	0.99	0.01	0.95	0.91	1.00	0.04	0.98	0.95	1.02	0.27	0.94	0.89	1.00	0.03
Fail or repeat exam in Y2	501	21	2.76	1.43	5.32	0.00	4.05	1.70	9.62	0.00	2.66	1.42	4.98	0.00	3.82	1.13	12.99	0.03
Fail or repeat exam in Y3	498	24	2.11	1.06	4.18	0.03	3.48	1.46	8.26	0.01	2.28	1.22	4.26	0.01	0.99	0.13	7.41	0.99

*UMAT* Undergraduate Medicine and Health Sciences test, *HSFY* Health Sciences First Year, *Int student* International students, *Y2* second year classes, *Y4* fourth year classes, *GPA* Grade Point Average for HSFY marks. Pathways that do not require UMAT include international, Maori and Pacific students and allied health graduates

## Discussion

For those students who were identified as having difficulties in their clinical attachments, we found that difficulties with clinical skills were just as prevalent as knowledge deficit. We assessed clinical skills by observing history taking and physical examination, and reading case histories. With regard to this, international students gaining direct entry into medical school without HSFY and UMAT-based selection were at highest risk. Students from ethnic minorities however were not at risk. We suggest there are two explanations for this: the first is that the entry criteria differ and rely mostly on GPA from the home university. UMAT paper 2, which we found may predict some clinical skills problems in many students, is not used as an entry criterion for international students. Secondly admission to medical school directly from their home country does not allow time for acculturation [2]. We did not quantitate the time interval between selection and direct entry into 2^nd^ or 4^th^ year classes, but we estimate this is only a matter of a few weeks. These students are at risk because they have insufficient time to master the challenges of communicating with, and adjusting to, the local peoples and culture. This is supported by our finding that students from minority ethnic groups, most of whom have been living in the country for a much longer time prior to selection, are not at risk despite English not being their first language.

By far the major contributor to clinical skills category was unsatisfactory history taking [as observed by an examiner] which was documented in 50% of students in the study group, though as the students progressed through to year 6 and acquired more clinical exposure to patients, the problem became less prevalent. We may underestimate how difficult history taking can be when the local language is unfamiliar to the student, and the patient is confused, deaf, stressed or uncooperative. When the student has to concentrate simultaneously on both language and clinical analysis, the task becomes much harder and important subtleties may be lost. In short, a major problem with comprehension or expression of English undermines the clinical power of history taking, the cornerstone of our diagnostic process [3, 16, 17]. When the language and local cultural nuances are mastered, other more advanced interview techniques can be employed, for example: establishing a rapport, being sensitive to the patient, clarifying ambiguous answers, framing questions appropriately, keeping the patient on track, establishing events in time, and processing the information as it is acquired [18, 19]. In an assessment situation, all of this has to be done “on the spot” and the student has to be able to compensate for the unexpected. It also requires of course, some clinical experience and medical knowledge to ask appropriate questions, and to know what parts of the history to amplify or discount.

It is interesting that other variables such as female gender, higher UMAT paper 2 scores, and graduate-entry predict a lower risk of clinical skills problems during clinical training. In view of public concern regarding fewer males entering medicine, it is reassuring to find that female students are less likely to have difficulty in their clinical training and no particular ethnic minority group is at risk [12, 20]. There is some evidence that women communicate better, are more “verbal” and perhaps more empathic in the interview situation [20]. Graduate entry students tend to be older, more mature and having had more time at University may have more background scientific knowledge, be more systematic in their approach, and therefore better able to deal with the challenges inherent in clinical medicine [21, 22]. Furthermore they have more time to acculturate before gaining entry. The beneficial effect of a higher mark in UMAT paper 2 (“understanding people”) lends some validity to the use of this measure as a selection criterion [6].

We also note that several predictive variables are associated with more than one problem category. We interpret this as indicating that the problems students face can often be interrelated. For example, a problem with professionalism could also be related to a problem with knowledge and the converse could also occur. While our problem categories are useful to help define the spectrum of issues faced, we do not suggest they are independent factors. This might also explain why UMAT paper 2 (‘understanding people’) predicted some professionalism problems.

The main limitation of our study is that we clustered problems into broad categories and adopted a sensitive, rather than specific approach to detection. This means we found a number of ‘false positives’ – students who subsequently performed well. Secondly our assessment of clinical skills was rather non-specific For example, when assessing history taking, it is often hard to dissect down to language problems, interview technique, or applied knowledge and, as most teachers are aware, several problems may co-exist in the same student. Furthermore, when language is deficient it is often very hard to assess interview technique and applied knowledge separately. As stated earlier, our results only relate to students at one clinical campus over a limited time. Our marking system is probably “over-sensitive” and we know that more than 90% of students identified subsequently met the conditions required and passed their summative assessments.

While most selection research has looked at predictors of success, we have looked at predictors of difficulty. We have found that some components of our current selection criteria are predictive for future difficulties with knowledge, professional behaviour and clinical skills, and that students who enter the course via different pathways are at greater risk during their clinical training. Our selection criteria would benefit from further evaluation to try to identify earlier ,those students who may have difficulties in the clinical setting.

## Conclusions

Using a sensitive detection system based on formal course assessments, we documented difficulties in 203/561 students during clinical training over a five year period.

For these students, we found that difficulties with clinical skills were just as prevalent as knowledge deficit.

International students were at highest risk for having difficulty and this probably related to different selection pathways and delayed acculturation.

Graduate entry, female sex, and better performance in UMAT paper 2 conferred a lower risk for having difficulties during clinical training.

## References

[1] Prideaux D, Roberts C, Eva K, Centeno A, McCrobie P, McManus C, Patterson F, Powis D, Tekian A, Wilkinson D (2011). Assessment for selection for the health care professions and specialty training: consensus statement and recommendations from the Ottawa 2010 conference. Med Teach.

[2] Sadlacek WE, Prieto DO (1990). Predicting minority students’ success in medical school. Acad Med.

[3] Simpson M, Buckman R, Stewart M, Maguire P, Lipkin M, Novack D, Till J (1991). Doctor-patient communication: the Toronto consensus statement. B MedJ.

[4] Siu E, Reiter H (2009). Overview: what’s worked and what hasn’t as a guide towards predictive admissions tool development. Adv in Health Sci Educ.

[5] Fergusson E, James D, Madley L (2002). Factors associated with success in medical school: systematic review of the literature. B MedJ.

[6] Poole P, Shulruf B, Rudland J, Wilkinson T (2012). Comparison of UMAT scores and GPA in prediction of performance in medical school: a national study. Medical Educ.

[7] Kreiter C, Kreiter Y (2007). A validity generalization perspective on the ability of undergraduate GPA and the medical college admission test to predict important outcomes. Teach Learn Med.

[8] Kreiter C, Yin P, Solow C (2004). Investigating the reliability of the medical schools admissions interview. Adv Health Sci Educ.

[9] Powis D, Waring T, Bristow T, O’Connell D (1992). The structured interview as a tool for predicting premature withdrawal from medical school. Aust NZ J Med.

[10] Winston KA, van der Vleuten CP, Scherpbier AJ (2014). Prediction and prevention of failure: an early intervention to assist at-risk medical students. Med Teach.

[11] Wilkinson TJ, Tweed M, Egan T, Ali A, McKenzie J, Moore M (2011). Joining the dots: Conditional pass and programmatic assessment enhances recognition of problems with professionalism and factors hampering student progress. BMC Med Educ.

[12] Chisholm D (2010). The disappearing white male doctor. North and South.

[13] Crampton P (2012). The challenges of selecting students. NZ Med J.

[14] Poole P, Moriaty H, Wearn A, Wilkinson T, Weller J (2009). Medical student selection in New Zealand: looking to the future. NZ Med J.

[15] Fontaine SMF, Wilkinson TJ (2003). Monitoring medical students’ professional attributes: Development of an instrument and process. Adv Health Sci Educ.

[16] Teutsch C (2003). Patient-doctor communication. Med Clin North Am.

[17] Woloshin S, Bickell NA, Schwartz LM, Gany F, Welch HG (1995). Language barriers in medicine in the United States. JAMA.

[18] Duffy FD, Gordon GH, Whelan G, Cole-Kelly K, Frankel R (2004). Assessing competence in communication and interpersonal skills: the Kalamzoo II report. Acad Med.

[19] Kalet A, Pugnaire MP, Cole-Kelly K, Janicik R, Ferrara E, Schwartz MD, Lipkin M, Lazare A (2004). Teaching communication in clinical clerkships: models from the Macy initiative in health communications. Acad Med.

[22] Elliott SL, Epstein J (2005). Selecting the future doctors: the role of the graduate medical programme. Int Med J.

[20] Lievens F (2013). Adjusting medical school admission: assessing interpersonal skills and situational judgement tests. Med Educ.

[21] Calvert MJ, Ross NM, Freemantle N, Yong XU, Renigio Z, Parle JV (2009). Examination of graduate entry medical students compared with mainstream students. J Royal Soc Med.

